# The Five Pillars of Acute Right Ventricular Heart Failure Therapy: Can We Keep the Pediment in Balance?

**DOI:** 10.3390/jcm13226949

**Published:** 2024-11-18

**Authors:** Antoniu Octavian Petriş, Călin Pop, Diana Carmen Cimpoeşu

**Affiliations:** 1 Cardiology Clinic, “Grigore T. Popa” University of Medicine and Pharmacy, 700115 Iaşi, Romania; antoniu.petris@yahoo.ro (A.O.P.); dcimpoiesu@yahoo.com (D.C.C.); 2 “Sf. Spiridon” Clinical County Emergency Hospital, 700111 Iaşi, Romania; 3 Faculty of Medicine, West “Vasile Goldiş” University, 310025 Arad, Romania; 4 Emergency Department, “Grigore T. Popa” University of Medicine and Pharmacy, 700115 Iaşi, Romania

**Keywords:** right heart failure, pulmonary embolism, cor pulmonale, pillar, ECMO

## Abstract

Acute right ventricular heart failure (aRHF), a long-neglected aspect of heart disease, has recently gained attention due to an improved understanding of its pathophysiology and the development of tailored therapeutic strategies. The therapeutic approach is now built on several pillars that aim to support the stable clinical condition of the patient, starting with the central pillar of etiological or specific therapy and extending to various aspects related to hemodynamic support, ventilation support, fluid optimization, and, when necessary, advanced resources such as right ventricular assist devices (e.g., extracorporeal membrane oxygenation—ECMO, Impella RP, or ProtekDuo). This five-pillar approach summarizes the different facets of contemporary treatment for aRHF, although some aspects related to their use are still being clarified.

## 1. Introduction

Long considered the “weak ventricle”, “a passive conduit”, “the Cinderella side of the heart”, or “a forgotten ventricle”, the right ventricle (RV) has recently demonstrated its importance, prompting contemporary investigations and re-evaluations that emphasize its role in maintaining normal heart function.

The RV was not only “forgotten” but also minimized regarding its role in ensuring a normal hemodynamic after early experiments (Starr et al. 1943), which promoted the idea that “a normal, contractile right ventricular wall is not necessary for the maintenance of a normal circulation”, a concept that determined the complete exclusion of the RV in Fontan operations (1971) [1]. It can be said that cardiologists have become more and more “LV-centric”. The end-diastolic volume of the RV is greater than that of the left ventricle/LV (49–101 mL/m^2^ vs. 44–89 mL/m^2^), its ejection fraction (45–60%) is less than that of the LV (50–70%) and its elastance (1.30 ± 0.84 mmHg/mL) is also more reduced than that of the LV (5.48 ± 1.23 mmHg/mL) [2]. More recently, the important prognostic role of RV dysfunction was proved in congenital heart disease (CHD), pulmonary hypertension (PH), and left heart failure (LHF). LV contraction generates up to 40% of “weak” RV contractile force, but this is sufficient to pump systemic venous return into the low-pressure system of pulmonary circulation. One of the greatest vulnerabilities of the RV is its greater sensitivity to changes in afterload [3].

Acute right heart failure (aRHF) can be isolated (characterized by increased RV and atrial pressure with systemic congestion) or may be associated with LV damage and reduced systemic cardiac output (due to ventricular interdependence, highlighting the importance of maintaining the transseptal gradient) [4].

Consequently, the etiology of aRHF primarily includes acute pulmonary embolism (aPE), acute cor pulmonale (in previously healthy individuals, high-risk aPE is the most common cause of acute RV failure) [5], acute right myocardial infarction (aRMI, in which the right coronary artery perfuses the RV free wall and the posterior part of the interventricular septum during both systole and diastole activity, making the RV more vulnerable to increases in wall tension and drops in systemic blood pressure) [6], acute respiratory distress syndrome (ARDS, with 20–30% of moderate to severe aRDS cases developing aRHF), acute-to-chronic cor pulmonale (a/cCP) [6], and aRHF post-cardiac surgery (pcsRHF) (in 0.1% of patients after cardiotomy, 2–3% after heart transplantation, and in 10–20% of cases in LV assisted device insertion) [7]. Pericardial pathology is not addressed in this work, although the pericardium participates in maintaining ventricular interdependence and does not readily accommodate acute changes in RV size in response to afterload variations [8].

**Figure 1:**
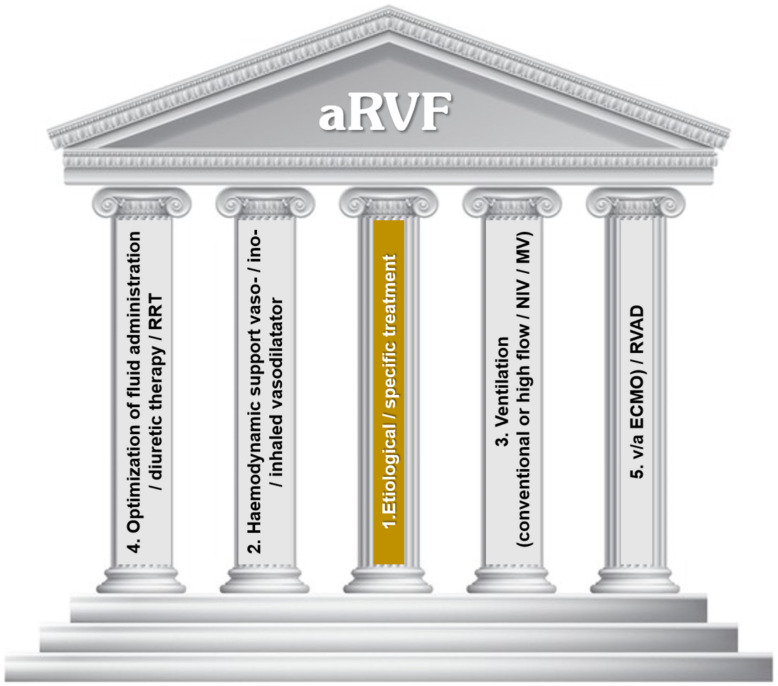
The five-pillar approach for the treatment of aRHF: (1) etiological/specific treatment; (2) hemodynamic support (vaso-/ino-/inhaled vasodilator); (3) ventilation (conventional or high flow/NIV/MV); (4) optimization of fluid administration/diuretic therapy/RRT; and (5) v/a ECMO)/RVAD. NIV—non-invasive ventilation. MV—mechanical ventilation; RRT—renal replacement therapy; v/a—ECMO veno/arterial extracorporeal membrane oxygenation; RVAD—right ventricular assist device; aRHF—acute right heart failure.

The management of aRHF typically begins in emergency settings, such as emergency departments and intensive cardiovascular care units, involving a multidisciplinary approach (emergency medicine, cardiology, pneumology, internal medicine, intensivists, and thoracic surgery) [5]. Since the *primum movens* in any emergency approach is its organization, in this work, we propose and verify the consistency of references regarding the establishment of some pillars on which such a therapeutic approach must be built, following the model recently used in LV heart failure (Figure 1). A major pathophysiological specificity of the RV is its heightened sensitivity to increases in afterload, regardless of origin. Accordingly, therapeutic approaches aimed at decreasing excessive RV afterload should be prioritized, while treatment of the underlying cause of RVF can be addressed subsequently. However, identifying the etiology and addressing it is the “gold” solution to interrupt the downward spiral of the patient’s clinical condition, with various symptomatic supports used merely to buy time until the main etiology is identified and removed. Of course, specific differences in management strategies may depend on the resources available in different countries and local diagnostic capabilities. Currently, much of what we know about pharmacological or interventional support in aRHF comes from various animal models (canine, porcine, rabbit, and rat), which exhibit differences among models and in local availability, thereby generating variability in the results [9], as well as from case reports, small cohorts, or meta-analysis. The need for randomized, controlled, multicenter studies is obvious. The pillars/columns of aRHF management are part of the therapeutic architecture by which the modern treatment of chronic heart failure, CKD, atrial fibrillation, etc. has recently been constructed. This seems to be an efficient way to highlight the structures that more robustly or more precariously support the condition of stability of the respective patient, allowing for personalized treatment. These management pillars follow the concept of some physiopathological pillars that the optimal approach to aRHF must take into account: primary etiology that must be removed as quickly and as completely as possible, contractility that must be enhanced, preload (volume) that will have to be optimized, post-load (pressure) that will have to be reduced, and secondary organ damage that will have to be prevented. It is important to note the fact that some therapeutic resources support several pillars at the same time or in sequence.

There are a series of therapeutic peculiarities in the case of patients who are older, pregnant, oncologic, etc., but their approach is beyond the scope of our paper.

## 2. The First Pillar

Since a decision-making hierarchy could improve the applicability of this framework, especially in emergency conditions, we believe that the first pillar, which is also the central pillar that can ensure stable support, should consist of the etiological/specific treatment, representing an essential target of the management of aRHF. The other pillars intended to support the pediment are added gradually, taking into account the advantages and limitations that each one has. This pillar is best supported by data from clinical trials of varying sizes (e.g., anticoagulation/systemic thrombolysis/percutaneous catheter-directed treatment/surgical pulmonary embolectomy in aPE, PCI/thrombolysis in aRMI, etc.) [10].

Frequently encountered challenges in emergency situations mainly relate to the availability of diagnostic and therapeutic resources because the therapeutic intervention will have to be applied as quickly as possible and must be adapted to the etiology. Sometimes it is challenging to discern if the RV dysfunction is secondary to left-sided cardiac dysfunction, a pulmonary pathology (airway, parenchymal, or vascular disease), an isolated right ventricle aRMI or pulmonary circulation failure (aPE), or a combination of these etiologies.

For the early and appropriate treatment of the underlying causes of aRHF (before RV dysfunction passes the point-of-no-return), diagnostic resources must be available in emergency settings, including a fast and focused clinical assessment, electrocardiography, biomarkers (D-dimers, natriuretic peptides, troponin, and blood lactate), echocardiography (at least a point-of-care ultrasound or POCUS), and other emergency imaging techniques. No specific biomarker has been identified for the early diagnosis of aRVF, but natriuretic peptides and cardiac troponin levels do have prognostic value. In acute pulmonary embolism, for example, levels of NT-proBNP ≥ 600 ng/L, heart-type fatty acid binding protein (H-FABP) ≥ 6 ng/mL, or copeptin ≥ 24 pmol/L may provide additional prognostic information, but these biomarkers have not yet been validated to guide treatment decisions [10]. Echocardiographic evaluation is the most frequently used technique in daily practice and several valuable parameters for an imaging diagnosis of aRVF have been identified: fractional area change (FAC), tricuspid annular plane systolic excursion (TAPSE), the Doppler tissue imaging-derived systolic S’ velocity of the tricuspid annulus, or the RV index of myocardial performance (RIMP), and, by using strain echocardiography, RV global and regional longitudinal shortening may be estimated [3].

Invasive hemodynamic parameters indicating the presence of aRVF include elevated central venous pressure (CVP > 15 mmHg), discordant right-to-left filling pressures (the right atrial pressure to pulmonary capillary wedge pressure ratio or RAP/PCWP of > 0.8), a low pulmonary artery pulsatility index (PAPi ≤ 1.85, which should be interpreted cautiously in severe tricuspid regurgitation), and a low RV stroke work index (<0.25–0.30 mmHg·L/m^2^) [4]. The RAP/PCWP (right atrial pressure to pulmonary capillary wedge pressure) ratio shows that lowering PCWP increases pulmonary artery compliance more than would be anticipated from a fall in pulmonary vascular resistance (PVR) alone [11]. A low PAPi (pulmonary artery pulsatility index) value, derived noninvasively by transthoracic echocardiography, is associated with the markers of right heart failure, RV dysfunction, and worse survival rates [11].

In a previously healthy individual, the most common cause of acute RV failure is suspected to be a high-risk aPE [5], but we must not ignore the possibility of an aRMI involving the RV, whether it is in association with an inferior myocardial infarction (10–50%) or possibly also with the anterior one (13%, according to necroptic studies) [12].

The recommendations from the latest ESC guidelines for the treatment of aPE [10] specify the sequence, recommendation classes, and level of evidence for each therapeutic method. It is recommended that anticoagulation treatment with unfractioned heparin (UHF), including a weight-adjusted bolus injection, should be initiated without delay in patients with high-risk PE (class of recommendation I; level of evidence C). Systemic thrombolytic therapy is recommended for high-risk PE (I, B), while surgical pulmonary embolectomy or percutaneous catheter-directed treatment (such as catheter-directed thrombolysis (CDT), ultrasound-assisted CDT (USCDT), pharmacomechanical CDT, and aspiration thrombectomy) is recommended for patients with high-risk PE, in whom thrombolysis is contraindicated or has failed (I, C). The inferior vena cava filter should only be used in patients with a clear contraindication for anticoagulation (IIa, C); in these cases, the filter should be removed as soon as possible, due to the significant risk of subsequent deep vein thrombosis [5,10]. The routine use of IVC filters is not recommended (III, A).

The management of aRMI, as stated in the latest version of the ESC guidelines on this subject, includes early reperfusion (PCI/thrombolysis), which can lead to rapid hemodynamic improvement, the avoidance of reducing right ventricular preload (i.e., nitrates and diuretics and opioid medications), and the correction of atrio-ventricular (AV) dyssynchrony and/or AV block, with rhythm sequencing if necessary [13].

In the 2021 ESC guidelines regarding the management of patients with isolated aRHF, only two therapeutic indications have the recommendation class and level of evidence mentioned: loop diuretics (Class I) and vasopressors and/or inotropes (Class IIb) [4].

## 3. The Second Pillar

The second pillar focuses on hemodynamic support (vasopressors, inotropes/lusitropes, or inhaled vasodilators). The rationale for using vasopressors as first-line therapy is driven by the need to maintain right coronary perfusion pressure. The presence of biventricular dysfunction may indicate the use of inotropes. It should be noted that in cardiogenic shock from the right, as opposed to cardiogenic shock that mainly involves the left ventricle, a “double hit phenomenon” has been described. The “double hit phenomenon”, described by Hrymak et al., refers to severely reduced organ perfusion (liver, renal, gut, etc.) in a right ventricular shock because of the high CVP associated with low systolic blood pressure, in contrast to what happens during a shock that mainly affects the LV, where the organ perfusion is maintained, because both the central venous pressure (CVP) and the systolic blood pressure are both low [2]. The clinical significance of this phenomenon is that after the “first hit”, represented by the damage to the RV (an acute increase in RV afterload or volume results in increased wall tension, septal shift due to transseptal gradient reduction, and tricuspid regurgitation), multiple intra-abdominal organ failure occurs (“double hit”), which must be constantly considered and treated appropriately [2].

Norepinephrine (0.1–0.5 mcg/kg/min) is a reasonable first choice [8], improving systemic hemodynamics (as a potent α1-receptor agonist with weaker β-receptor activity, noradrenaline increases systemic arterial vascular resistance and increases cardiac output through the optimization of cardiac preload and direct inotropism, and also increases systemic blood pressure and coronary artery perfusion) with minimal effect on pulmonary vascular resistance [14]. Vasopressin (0.03 units/min) and vasopressin analogs may be useful as adjunct vasopressors if hypotension persists [10]. Inotropes can be used alone in cases of hypoperfusion without hypotension [4]. Inodilators (inotropes with vasodilatory properties) such as dobutamine, levosimendan, and phosphodiesterase-III inhibitors can reduce cardiac filling pressures, enhance ventriculo-atrial coupling by increasing RV contractility, and restore cardiac output, reducing afterload due to PA vasodilation; however, as inotropic agents may exacerbate arterial hypotension, they may be combined with norepinephrine if necessary [4,10]. Sympathomimetics, including norepinephrine and phenylephrine (the second is not as beneficial in studies on humans compared to those performed on animals), have a direct vasoconstrictive effect on the pulmonary artery, a property not shared by vasopressin [8].

In a/cCP, inotropic-vasoactive drugs and inhaled vasodilators have been proposed during awake intubation following the nebulization of local anesthesia. The simplest pulmonary vasodilator is supplemental oxygen [10], but the addition of inhaled nitric oxide (iNO, iloprost, or epoprostenol) is reasonable as a rescue therapy in patients with ongoing RV dysfunction despite hemodynamic support, appropriate volume status, and supplemental oxygen administration [5,10]. These therapies remain off-label and can increase ventilation/perfusion mismatch or shunting, worsening oxygenation [5]. Some experts argue that pulmonary vasodilators should not be classified as “hemodynamic support” but rather as strategies to decrease RV afterload.

## 4. The Third Pillar

The third pillar consists of ventilation support using oxygen therapy (either conventionally or via a high-flow nasal cannula, targeting an oxygen saturation of > 90%) [10], non-invasive ventilation (NIV), or mechanical ventilation (MV). Supplemental oxygen should be considered even without hypoxemia [8]. However, while MV may occasionally be required in the management of patients with aRHF (e.g., severe hypoxemia, impaired mentation, or the facilitation of procedures), it should ideally be avoided, particularly in the case of positive pressure ventilation, due to potential hemodynamic consequences, MV being associated with a three-fold higher risk of mortality in high-risk PE or a/cCP [15]. Pulmonary vascular resistance and intrathoracic pressures may increase, and venous return and RV preload may decrease during positive pressure ventilation; furthermore, the option to use positive end-expiratory pressure (PEEP) extends these effects throughout the respiratory cycle, further reducing venous return and RV preload [5]. Therefore, ventilation support in patients with aRVF may worsen the clinical situation and should be avoided in this clinical setting; when necessary, it should be used with caution (ultraprotective settings). Caution is advised regarding the risk of peri-intubation hemodynamic collapse (e.g., with propofol) and pulmonary vasoconstriction (worsening hypoxemia and hypercarbia during induction), with etomidate considered the most hemodynamically neutral induction agent [10]. Hypercarbia may not only occur after induction but can also be present depending on the severity of lung disease when RVF is associated with respiratory compromise. If MV is necessary, it should be approached cautiously with positive end-expiratory pressure and low to moderate tidal volumes; however, correcting hypoxemia may not always be feasible without simultaneous pulmonary reperfusion in high-risk aPE [10]. Early prone positioning is one of the best maneuvers for unloading the RV in aRDS [16].

## 5. The Fourth Pillar

The fourth pillar relates to the optimization of intravenous fluid (IVF) administration [5], the use of diuretic therapy (the potential benefits of IVF versus fluid removal depend on the baseline status of the patient with aRHF, with loop diuretics remaining the first option in cases of venous congestion; assessing volume responsiveness may be useful) [4,10] or, in some cases, the use of renal replacement therapy (RRT) [4]. A randomized open-label study comparing diuresis with a 0.5-L saline infusion reported a more rapid decrease in natriuretic peptide levels after diuretics, without any difference in RV function or clinical outcomes [17]. Thus, in most cases, IVF administration may be harmful (over-distending the right heart and subsequently increasing wall tension, impairing LV filling, aggravating tricuspid regurgitation, worsening ventricular interdependence, and, consequently, reducing cardiac output) [5]. Although the presentation of fluid optimization with diuretic therapy can be somewhat confusing, the evidence suggests that patients with aRVF usually do not require fluid supplementation, as this may exacerbate the associated systemic venous congestion, sometimes necessitating the removal of excess fluid. Recent contributions emphasize the possibility of false positive indices of fluid responsiveness, such as pulse pressure variation [18]. We need to be careful with volume loading that is guided by central venous pressure (CVP) monitoring, but a CVP of less than 10 mm Hg can almost rule out RV dysfunction with congestion [3]. In patients with ARDS, fluid responsiveness can be predicted, but different thresholds should be used [19]. IV loop diuretic administration should be considered, particularly if evidence exists of RV dysfunction or volume overload [10]. The challenge lies in detecting hypovolemia, which could be prognostic, while avoiding worsening tissue hypoperfusion in patients with systemic venous congestion. This remains an important and complex dilemma.

## 6. The Fifth Pillar

The fifth pillar represents the most modern approach: the use of veno/arterial extracorporeal membrane oxygenation (v/aECMO) and right ventricular assist devices (RVAD). Where both pulmonary and cardiac support are necessary, v/aECMO is the preferred approach [20]; for pulmonary insufficiency, v/vECMO can be considered (even in the absence of MV) as a bridge to an intervention in cases of progressive RV failure, but not in aPE, as it returns blood to the venous system and does not decrease RV preload [21]. When only cardiac support is needed, options such as Impella RP or ProtekDuo support (but not an intra-aortic balloon pump for isolated aRHF) should be considered. The choice of device depends on the estimated duration of mechanical RV support: short-term (10 to 15 days) devices include ECMO, Impella RP, and PROTEK Duo, while for more than 15 days, surgically implanted devices (Levitronix CentriMag) should be chosen, and, for an assisted VD in which recovery is not expected, the possibility of accessing a heart or heart-lung transplant should be assessed [22]. The association of therapeutic options is also crucial: in 39 studies (n = 6409) involving ECMO for aPE, patients treated with ECMO and catheter-directed therapy had significantly lower mortality compared to those treated with ECMO and systemic thrombolysis [23]. ECMO in high-risk PE and aRVF (refractory cardiac shock or cardiac arrest) cases, in combination with surgical embolectomy or catheter-directed treatment, represents a promising approach. It must be taken into account, as a warning, that ECMO might also induce adverse effects such as a reduction in bronchial arterial blood flow, a reduction in pulmonary blood flow/transpulmonary gradient, and the worsening of lung ischemia [24]. Based on the data currently available, we can state that the potential contraindications and procedural risks of right ventricular assist devices for Impella RP are the lack of an intrinsic oxygenator and a higher risk of hemolysis, for Protek Duo, they are the long insertion time and the high transfusion rate, and for v/a ECMO, they are LV distension and vascular complications [20]. Cost, accessibility, and training requirements remain a continuing challenge for the development of these forms of advanced mechanical support.

## 7. Conclusions

Acute *cor pulmonale* is a life-threatening entity in which many organs are affected; therefore, it requires a multidisciplinary approach [5]. The therapeutic arsenal for patients with aRHF, like the treatment of heart failure in general, has recently been enriched by a combination of established, newly developed, and re-evaluated drugs, as well as new support devices for the RV. We propose a new architectural framework for a therapeutic approach to aRHF therapy based on five pillars, which would simplify the selection of options in emergency situations and would make sure that none are overlooked. Of course, these five pillars vary in robustness and importance and are based on statistical arguments of varying strengths. This architectural approach prompts the identification of the most appropriate therapeutic resources, adapted to each patient. The wise use of widely available therapeutic resources will have to be supported by increasing availability and access to modern therapeutic resources, as selected by a Heart Team that can be activated in critical moments (e.g., PERT in pulmonary embolism, etc.). If the identification of the etiology is the basis of specific assurances of the functionality of the first pillar, starting from the second pillar (hemodynamic support), there are often antagonistic therapeutic features (the use of vasopressors, inotropes/lusitropes, or, in some situations, inhaled vasodilators); the cautious use of positive end-expiratory pressure with the option of low to moderate tidal volumes (third pillar), the cautious administration of fluids but also recourse to diuretic therapy (fourth pillar), and recourse to ECMO should be made as early as possible, but, depending on the need for cardiac support, Impella RP and PROTEK Duo may prove much more useful. The motto of this pediment-supporting effort might be “*Nec plus ultra*”—nothing further beyond.

## 8. Future Directions

In the future, *Artificial intelligence* (AI)-enhanced diagnostic and therapeutic strategies may improve the assessment of aRHF and provide optimal management in this acute setting. Progress can be achieved through early diagnosis with the identification of the underlying etiology (specific biomarkers for RV failure, which are commonly used in the current practice of molecular imaging, as well as the identification and use of new hemodynamic indices) and early treatment, even borrowing some effective drugs for LV heart failure (e.g., dapagliflozin for structural RV remodeling and antiarrhythmic effects), or reducing device implantation delays [22]. SGLT2i may improve RV function, based on TAPSE, PAP, and FAC values recorded in HF patients assessed with the CardioMEMS sensor, which showed that empagliflozin caused rapid decreases in PAP, independent of the loop diuretic effect [25,26].

## Data Availability

Data sharing is not applicable to this article as no datasets were generated or analyzed during the current outline of advances in our field of expertise.

## References

[1] Sanz J., Sánchez-Quintana D., Bossone E., Bogaard H.J., Naeije R. (2019). Anatomy, function, and dysfunction of the right ventricle: JACC State-of-the-Art Review. J. Am. Coll. Cardiol..

[2] Hrymak C., Strumpher J., Jacobsohn E. (2017). Acute right ventricle failure in the Intensive Care Unit: Assessment and management. Can. J. Cardiol..

[3] Harjola V.-P., Mebazaa A., Čelutkienė J., Bettex D., Bueno H., Chioncel O., Crespo-Leiro M.G., Falk V., Filippatos G., Gibbs S. (2016). Contemporary management of acute right ventricular failure: A statement from the Heart Failure Association and the Working Group on Pulmonary Circulation and Right Ventricular Function of the European Society of Cardiology. Eur. J. Heart Fail..

[4] McDonagh T.A., Metra M., Adamo M., Gardner R.S., Baumbach A., Böhm M., Burri H., Butler J., Čelutkienė J., Chioncel O. (2021). 2021 ESC Guidelines for the diagnosis and treatment of acute and chronic heart failure. Eur. Heart. J..

[5] Arrigo M., Price S., Harjola V.-P., Huber L.C., Schaubroeck H.A.I., Vieillard-Baron A., Mebazaa A., Masip J. (2024). Diagnosis and treatment of right ventricular failure secondary to acutely increased right ventricular afterload (acute cor pulmonale). A clinical consensus statement of the Association for Acute CardioVascular Care (ACVC) of the ESC. Eur. Heart J. Acute Cardiovasc. Care..

[6] Houston B.A., Brittain E.L., Tedford R.J. (2023). Right ventricular failure. N. Engl. J. Med..

[7] Kaul T., Fields B.L. (2000). Postoperative acute refractory right ventricular failure: Incidence, pathogenesis, management and prognosis. Cardiovasc. Surg..

[8] McGuire W.C., Sullivan L., Odish M.F., Desai B., Morris T.A., Fernandes T.M. (2024). Management strategies for acute pulmonary embolism in the ICU. Chest.

[9] Andersen A., van der Feen D.E., Andersen S., Schultz J.G., Hansmann G., Bogaard H.J. (2020). Animal models of right heart failure. Cardiovasc. Diagn. Ther..

[10] Konstantinides S.V., Meyer G., Becattini C., Bueno H., Geersing G.-J., Harjola V.-P., Huisman M.V., Humbert M., Jennings C.S., Jiménez D. (2020). 2019 ESC guidelines for the diagnosis and management of acute pulmonary embolism developed in collaboration with the European Respiratory Society (ERS). Eur. Heart J..

[11] Konstam M.A., Kiernan M.S., Bernstein D., Bozkurt B., Jacob M., Kapur K., Kociol R.D., Lewis E.F., Mehra M.R., Pagani F.D. (2018). Evaluation and management of right-sided heart failure. A scientific statement from the American Heart Association. Circulation.

[12] Femia G., French J.K., Juergens C., Leung D., Lo S. (2021). Right ventricular myocardial infarction: Pathophysiology, clinical implications and management. Rev. Cardiovasc. Med..

[13] Steg P.G., James S.K., Atar D., Badano L.P., Blömstrom-Lundqvist C., Borger M.A., Di Mario C., Dickstein K., Ducrocq G., Fernandez-Aviles F. (2012). Task Force on the management of ST-segment elevation acute myocardial infarction of the European Society of Cardiology (ESC). ESC Guidelines for the management of acute myocardial infarction in patients presenting with ST-segment elevation. Eur. Heart J..

[14] Legrand M., Zarbock A. (2022). Ten tips to optimize vasopressors use in the critically ill patient with hypotension. Intensive Care Med..

[15] Disselkamp M., Adkins D., Pandey S., Yataco A.O.C. (2018). Physiologic approach to mechanical ventilation in right ventricular failure. Ann. Am. Thorac. Soc..

[16] Guerin C., Beuret P., Constantin J.M., Bellani G., Garcia-Olivares P., Roca O., Meertens J.H., Maia P.A., Becher T., Peterson J. (2018). A prospective international observational prevalence study on prone positioning of ARDS patients: The APRONET (ARDS Prone Position Network) study. Intensive Care Med..

[17] Ferrari E., Sartre B., Labbaoui M., Heme N., Asarisi F., Redjimi N., Fourrier E., Squara F., Bun S., Berkane N. (2022). Diuretics versus volume expansion in the initial management of acute intermediate high-risk pulmonary embolism. Lung.

[18] Vieillard-Baron A., Prigent A., Repessé X., Goudelin M., Prat G., Evrard B., Charron C., Vignon P., Geri G. (2020). Right ventricular failure in septic shock: Characterization, incidence and impact on fluid responsiveness. Crit. Care..

[19] Joseph A., Evrard B., Petit M., Prat G., Slama M., Charron C., Vignon P., Goudelin M., Vieillard-Baron A. (2024). Fluid responsiveness in acute respiratory distress syndrome patients: A post hoc analysis of the HEMOPRED study. Intensive Care Med..

[20] Kadri A.N., Alrawashdeh R., Soufi M.K., Elder A.J., Elder Z., Mohamad T., Gnall E., Elder M. (2024). Mechanical support in high-risk pulmonary embolism: Review article. J. Clin. Med..

[21] Götzinger F., Lauder L., Sharp A.S.P., Lang I.M., Rosenkranz S., Konstantinides S., Edelman E.R., Böhm M., Jaber W., Mahfoud F. (2023). Interventional therapies for pulmonary embolism. Nat. Rev. Cardiol..

[22] Monteagudo-Vela M., Tindale A., Monguió Santín E., Reyes-Copa G., Panoulas V. (2023). Right ventricular failure: Current strategies and future development. Front. Cardiovasc. Med..

[23] Boey J.J.E., Dhundi U., Ling R.R., Chiew J.K., Fong N.C.-J., Chen Y., Hobohm L., Nair P., Lorusso R., MacLaren G. (2024). Extracorporeal membrane oxygenation for pulmonary embolism: A systematic review and meta-analysis. J. Clin. Med..

[24] Helms J., Carrier M., Klok F.A. (2023). High-risk pulmonary embolism in the intensive care unit. Intensive Care Med..

[25] Mariani M.V., Manzi G., Pierucci N., Laviola D., Piro A., D’Amato A., Filomena D., Matteucci A., Severino P., Miraldi F. (2024). SGLT2i effect on atrial fibrillation: A network meta-analysis of randomized controlled trials. J. Cardiovasc. Electrophysiol..

[26] Tufan Cinar T., Saylik F., Cicek V., Pay L., Khachatryan A., Alejandro J., Erdem A., Hayiroglu M.I. (2024). Effects of SGLT2 inhibitors on right ventricular function in heart failure patients: Updated meta-analysis of the current literature. Kardiol. Pol..

